# The Effects of a Distracting *N*-Back Task on Recognition Memory Are Reduced by Negative Emotional Intensity

**DOI:** 10.1371/journal.pone.0110211

**Published:** 2014-10-16

**Authors:** Luciano G. Buratto, Claire L. Pottage, Charity Brown, Catriona M. Morrison, Alexandre Schaefer

**Affiliations:** 1 Department of Psychology, Durham University, Durham, United Kingdom; 2 Department of Psychology, University of Leeds, Leeds, United Kingdom; 3 School of Business, Monash University Malaysia, Bandar Sunway, Malaysia; 4 Department of Psychology, Heriot-Watt University, Edinburgh, United Kingdom; The University of Chicago, United States of America

## Abstract

Memory performance is usually impaired when participants have to encode information while performing a concurrent task. Recent studies using recall tasks have found that emotional items are more resistant to such cognitive depletion effects than non-emotional items. However, when recognition tasks are used, the same effect is more elusive as recent recognition studies have obtained contradictory results. In two experiments, we provide evidence that negative emotional content can reliably reduce the effects of cognitive depletion on recognition memory only if stimuli with high levels of emotional intensity are used. In particular, we found that recognition performance for realistic pictures was impaired by a secondary 3-back working memory task during encoding if stimuli were emotionally neutral or had moderate levels of negative emotionality. In contrast, when negative pictures with high levels of emotional intensity were used, the detrimental effects of the secondary task were significantly attenuated.

## Introduction

A wealth of research has demonstrated that encoding information in memory while depleting cognitive resources by a concurrent task leads to a decrease in subsequent memory [1], [2]. This cognitive depletion effect is usually found when contrasting memory performance associated with two distinct encoding tasks: a divided attention condition, during which a concurrent task has to be performed while items are studied, and a full attention condition, which does not involve a concurrent task. Studies using recall tasks to measure memory performance have found that memory costs due to divided attention are smaller for emotional stimuli than for neutral stimuli [3], [4]. These results suggest that emotional content can moderate the effects of cognitive depletion on recall. It is less clear, however, whether similar effects can also be obtained in recognition tasks.

For instance, Clark-Foos and Marsh [5] showed that recognition accuracy was higher for negative words than for neutral words under both full and divided attention conditions. These results showed that the emotional enhancement of memory survived the restriction of cognitive resources at encoding. However, these authors have not found a significant interaction between encoding task and stimulus emotionality, suggesting that emotional content did not moderate cognitive depletion effects in their study. Other studies combining a divided attention paradigm with recognition tests for emotional stimuli also reported no interaction between emotional content and encoding task (full vs. divided attention), using both word and face stimuli [6], [7].

By contrast, Kensinger and Corkin [8], although not explicitly addressing this issue, reported descriptive statistics suggesting that high-arousal words may have been less affected by cognitive depletion than low-arousal words. Maddox and colleagues [9] also recently reported an interaction between emotional content and encoding task in a recognition task. These authors found that word recognition performance was overall higher for negative compared to both positive and neutral words, and that this effect was stronger under divided attention. In addition, the figures reported by Maddox et al. [9] suggest that negative stimuli were less affected by divided attention than neutral or positive stimuli. These contradictory results, and in particular the descriptive statistics of Kensinger & Corkin (2004), could suggest that the effects of cognitive depletion on recognition are moderated only by stimuli with high levels of emotional intensity. However, this hypothesis has to our knowledge never been formally tested.

It is thus still open to debate whether or not emotional stimuli are more resilient against the deleterious effects of cognitive depletion on recognition memory. Furthermore, two features of these previous studies may have contributed to the conflicting results. First, most of these studies have operationalized emotion as a simple factor contrasting emotional vs. neutral items without taking into account that emotional stimuli can be differentiated according to their levels of *emotional intensity* (i.e., the perceived strength of an emotional reaction to a stimulus). In other words, these studies did not break down emotional stimuli into categories of “high”, “medium” or “low” intensity. Instead, all of these categories are typically merged into a single “emotional” (or “negative’ or “positive”) category. Therefore, if a protective effect against memory depletion is due to contents of higher emotional intensity, as suggested by Kensinger & Corkin's (2004) descriptive statistics, then this effect might have been masked by the absence of a differentiation between high and low intensity emotional stimuli in most previous studies. This hypothesis would be consistent with evidence that emotional content can have an effect on memory that varies according to the levels of intensity of the emotional stimuli employed [10], [11].

The second possible reason for the discrepancies in previous studies was the use of relatively short retention intervals (from seconds to minutes). In recognition memory tasks, the effects of emotion on recognition performance tend to be robust and stable if long (at least several hours) study-test intervals are used [12]–[14]. These delayed effects of emotional content on recognition memory are likely due to the long time-course of action of emotion-related hormones and neurotransmitters [15]. Because most previous studies investigating the effects of divided attention on the recognition of emotional information did not use long retention intervals, they may not have enabled a fair test of the long-term impact of emotion on divided attention costs.

We report here two experiments that provide evidence that negative emotional content can reliably reduce the effects of cognitive depletion on recognition performance only if highly intense emotional items are used. We used realistic emotionally negative and neutral pictures clearly differentiated in three levels of emotional intensity, defined here as the perceived emotional strength of a given stimulus. In addition, we used longer retention intervals than in previous recognition studies (4 hours in Experiment 1 and 2 days in Experiment 2) to maximize the chances of observing a reliable effect of emotional content on memory. Cognitive depletion was implemented with a 3-Back vs. 0-Back number working memory task while participants had to encode emotional and neutral pictures (referred to hereafter as the “secondary task”). This procedure is known to be very effective in imposing high demands on cognitive resources [16]–[19]. In addition, this procedure is ideal to examine the effects of cognitive load on picture processing while maintaining constant viewing conditions across divided and full attention conditions [20].

We hypothesized that, if high levels of emotional intensity have a unique role in protecting recognition memory from the deleterious effects of cognitive depletion, then only high-intensity negative stimuli should remain unaffected by dividing attention at encoding.

## Experiment 1

### Method

#### Participants

Forty adults (*M_age_*  = 24.3, *SD*  = 3.5; 12 males) took part in this experiment. Participants were paid £10 and evenly assigned to each N-Back condition. Two participants (one from each condition) with hit rate scores significantly deviating from their group average (*Z* = +/−2.5) were deemed as outliers and excluded from the recognition data. All participants signed an informed consent and the study was approved by the Ethics Committee of the Institute of Psychological Sciences at the University of Leeds.

#### Stimuli and Design

A total of 240 images (120 negative and 120 neutral) taken from IAPS [21] and Google Images were used. All images were previously rated for valence and arousal using a 5-point version (Valence: 1 =  negative, 5 =  positive; Arousal: 1 =  low, 5 =  high) of the Self-Assessment Manikin [SAM; 22]. These ratings were obtained from a sample of 51 British students from the University of Leeds (UK). Each picture was rated by a minimum of 9 participants and a maximum of 21 participants [23]. The stimuli used in both Experiments 1 and 2 were selected from a larger pool of 961 pictures rated following the procedure described above. The stimuli were split into two lists (A and B) containing 120 images each. These lists were used as sets of *Old* (studied) or *New* (unstudied) images in a counterbalanced manner across participants. Within each list (A or B), 60 of the images were negative and 60 were neutral. Similar to previous research [10], [24], we used the picture arousal scores to create different stimuli sets of emotional intensity. The neutral pictures formed the low intensity condition (*Low*) as they had a homogenous and low level of arousal, consistent with previous research [10], [24]. Next, the set of negative pictures was further subdivided into two groups of 30 high-intensity (*High*) and 30 medium-intensity pictures (*Medium*) by a median split on their arousal ratings. Picture groups differed in valence [*F*(2,237)  = 480.58, *P<*.001, 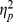  = .80] and arousal ratings [*F*(2,237)  = 346.93, *P<*.001, 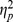  = .75]. All pairwise comparisons were significant (*Ps <*.001). Mean valence and arousal ratings are summarized in Table 1.

**Table 1:** Stimulus properties by Picture Type in Experiment 1.

	Picture Type					
	High		Medium		Low	
Property	*M*	*SD*	*M*	*SD*	*M*	*SD*
Valence	1.78	0.38	2.32	0.23	3.26	0.32
Arousal	3.60	0.39	2.76	0.29	2.11	0.40
Brightness	105.21	30.37	97.48	32.46	98.38	31.71
Contrast	13.62	6.16	15.44	7.08	13.28	6.80
Spatial freq.	16.85	10.39	18.29	11.48	19.83	11.74

*Note.* High  =  High emotional intensity; Medium  =  Medium emotional intensity; Low  =  Low emotional intensity (neutral).

As in other studies [23], [25], pictures obtained from “Google Images” were added to ensure that negative and neutral pictures were matched in relevant non-emotional dimensions (presence of humans, human faces, animals and objects), and lists A and B were also equated on these dimensions. In addition, low-level image properties (brightness, contrast, and spatial frequency) were matched across picture groups (High, Medium, Low), following the approach used by Bradley et al. [26]. More specifically, picture properties (brightness, contrast and spatial frequency) were extracted for each picture using MATLAB. Brightness was defined as the mean red, green and blue intensity for each pixel averaged across all pixels in the picture. Contrast was obtained in two steps: First, the standard deviation of pixel intensities in each image column was computed; then the standard deviation across all image columns was computed. The latter was used as an index of contrast. Spatial frequency was obtained in three steps: First a power spectrum of the image was computed; then the frequency that split the area under the power spectrum in two equal halves was obtained for each row and for each column of the image; finally, these median-split frequencies were averaged across all rows and columns. This average was used as an index of the dominant spatial frequency in the picture. Negative and neutral pictures did not differ significantly in any of these measures (all *P*s>.12).

Stimuli in the secondary task were numbers ranging from 1 to 28. In the 0-Back task, participants were asked to press one of two buttons if the number on display was a “5”. In the 3-Back task, participants were asked to press one of two buttons if the number on display was the same as the number displayed three trials earlier. Picture type was manipulated within subjects (High vs. Medium vs. Low) and secondary task was manipulated between subjects (0-Back vs. 3-Back).

#### Procedure

The experiment was divided into an encoding stage and a retrieval stage, separated by an interval of 4 hours. At encoding, participants viewed the pictures of one of the sets (A or B, counterbalanced across subjects) displayed on a 17″ screen, using E-Prime. Emotional and neutral pictures were intermixed, and presentation order was randomized. For every trial, a fixation cross appeared for 500 ms, followed by an emotional or neutral image, displayed for 2000 ms, followed by a black digit on a white background, displayed for up to 5000 ms. Participants in the 0-Back condition had to decide with a key-press whether or not the digit on display was a “5” (0-Back condition) or whether or not the number currently displayed matched the number displayed three trials earlier (3-Back condition). The trial terminated after a key-press or after 5000 ms had elapsed. For both conditions, participants were simply instructed to watch the pictures as they were displayed on the screen. There were three blocks of 40 trials with a brief rest period between each block, and two different lists of digits were used and fully counterbalanced across participants. Before the experiment, participants were given 0-Back and 3-Back practice trials to get familiarized with the procedure.

Four hours after the start of the study phase, participants performed a surprise recognition test in which they viewed all the pictures (sets A and B) in a random order, with “old” and “new” items intermixed. For every test trial, a fixation cross appeared for 2000 ms, followed by an emotional or neutral image displayed for 2000 ms. Next, a screen prompted participants to perform an old/new discrimination using a keyboard press. The old/new prompt was presented in the form of a 6-point confidence scale (from 1 =  *absolutely sure new* to 6 =  *absolutely sure old*). For each trial in which participants responded “Old”, a Remember–Know judgement task was required [27], [28]. Subjects were given the following instructions: “You may recognize the picture very vividly and remember specific details about its previous occurrence. In other words, a remember response implies that you recall something specific that happened when you first encountered this picture, during the first session of this experiment. This may include recalling what you thought when you first encountered this picture, or anything else that happened when you first saw this picture. When that is the case, choose the “Remember” option. However, you may just have a feeling of knowing the picture, when you cannot recall specific details about its first occurrence, but simply know that you have seen it before. When that is the case, choose the “Know” option”. Subjects made the Remember–Know judgments using keyboard presses.

#### Participant-level analyses

Memory data was analysed using standard recognition measures (hit rates, false-alarm rates, and *Pr*). Hit rate (*HR*) is the proportion of “old” responses (options “4”, “5”, or “6” in the 6-point confidence scale) given to studied (*Old*) pictures. False-alarm rate (*FAR*) is the proportion of “old” responses to unstudied (*New*) pictures. *Pr* is the difference between hit rates and false-alarm rates (*Pr*  =  *HR* – *FAR*). Data was analyzed with a mixed-design ANOVA (Analysis of Variance), with Picture Type (High, Medium, Low) entered as a within-subjects factor and Secondary Task (0-Back, 3-Back) entered as a between-subjects factor. Given our research question, we hypothesized that, if emotional intensity at encoding modulates the effects of the secondary task on recognition memory, then we should observe a Picture Type × Secondary Task interaction. Separate HR and FAR were also analysed for “Remember” (R) and “Know” (K) responses. R responses can be interpreted as an estimate of recollection processes at work during recognition, whereas K responses can be taken as estimates of familiarity in the absence of recollection [29], [30]. In the analyses reported in this study, we used corrected K responses (*K_cor_*), which take into account the number of R responses produced by the participant and is given by *K_cor_*  =  *K*/(1–*R*), where *K* is the proportion of K responses (K-HR if the response is given to an *Old* picture, K-FAR if given to a *New* picture) and *R* is the proportion of R responses (R-HR if the response is given to an *Old* picture, R-FAR if given to a *New* picture). We also analysed confidence judgements. However, given that the results obtained on confidence judgments did not significantly differ from the results obtained from the other recognition parameters, we have included them in the supplementary section (File S1) for the sake of conciseness.

#### Stimulus-level analyses

The analyses above allow to test the main hypotheses following a classical approach. However, they do not allow to directly contrast the size of the memory depletion effect between different conditions of emotional intensity. Further, they do not allow to control for low-level stimulus properties. Recent research suggests that low-level physical properties of pictorial stimuli may co-vary with picture emotionality [26]. Therefore, we also ran an ANCOVA (Analysis of Covariance) using each picture as the main unit of analysis. This analysis tested the effects of Picture Type on the magnitude of the N-Back-driven depletion of recognition memory, while controlling for low-level picture properties (brightness, contrast and spatial frequency). Here the effect of cognitive depletion was estimated for each picture by a difference score between the mean *Pr* discrimination score for this picture in the 0-Back and the 3-Back conditions. We then computed a one-way ANCOVA testing the effects of Picture Type with brightness, contrast and spatial frequency entered as covariates.

### Results

#### Recognition data: Hits, false alarms and Pr

A 3 (Picture Type: High, Medium, Low) ×2 (Secondary Task: 0-Back, 3-Back) mixed-design ANOVA on hit rates revealed main effects of Picture Type [*F*(2,72)  = 81.79, *P<*.001, 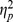  = .69] and Secondary Task [*F*(1,36)  = 16.15, *P<*.001, 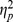  = .31]. Pairwise contrasts yielded significant differences between High and Medium intensity pictures (*M_HR_*  = .87,.72, *SE*s  = .01,.02, respectively) and between Medium and Low intensity pictures (*M_HR_*  = .63, *SE*  = .02; *Ps <*.001). Hit rates were also higher in the 0-Back compared to the 3-Back condition (*M_HR_*  = .80,.67, *SE*s  = .02). Importantly, the interaction was significant [*F*(2,72)  = 5.93, *P<*.01, 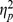  = .14]. Planned contrasts revealed that the difference in HR between the N-Back tasks was greater for Medium and Low intensity pictures (*t*s>3.8, *P*s ≤.001, Cohen's *d*s*>* 1.24, large effect) than for High intensity pictures (*t* = 2.1, *P = *.04, *d* = 0.68, medium effect). These results indicate that the 3-Back task reduced correct recognition of Medium and Low intensity pictures more than for High intensity pictures.

For false-alarm data, only a main effect of Picture Type [*F*(2,72)  = 7.12, *P<*.01, 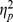  = .17] was found, driven by lower error rates for High intensity pictures compared to Medium and Low intensity pictures (*M_FAR_*  = .10,.14,.14, *SE*s  = .02, *Ps <*.01). Table 2 summarizes the results for hits and false alarms.

**Table 2:** Proportion of “Old” responses in the recognition memory test as a function of Picture Type, Picture Status and Secondary Task (Experiment 1).

		Picture Type					
N-Back task	Picture status	High		Medium		Low	
		*M*	*SD*	*M*	*SD*	*M*	*SD*
0-Back	Old	.89	.09	.80	.14	.72	.17
	New	.08	.11	.11	.11	.12	.08
3-Back	Old	.83	.08	.64	.10	.54	.10
	New	.11	.10	.17	.13	.17	.12

*Note. Hit rate*  =  Proportion of “Old” responses to *Old* pictures; *False-alarm rate*  =  Proportion of “Old” responses to *New* pictures. High  =  High emotional intensity; Medium  =  Medium emotional intensity; Low  =  Low emotional intensity (neutral).

**Figure 1:**
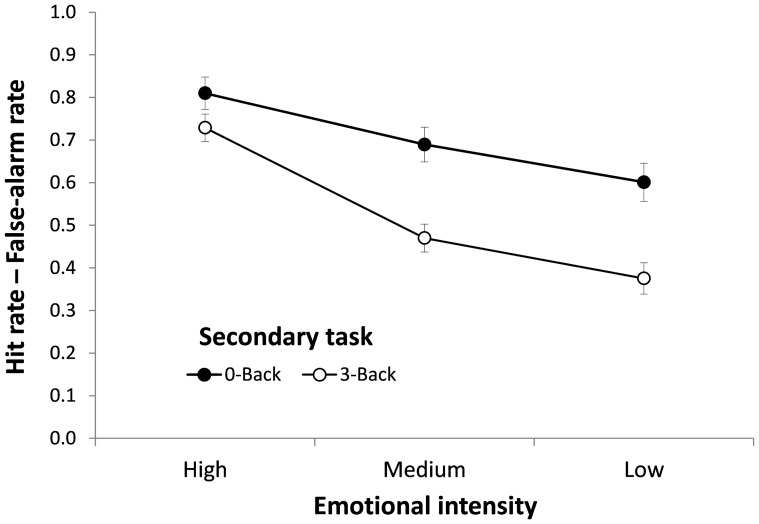
Recognition memory as a function of Picture Type and Secondary Task (Experiment 1). Memory performance was measured with *Pr*, the difference between hit rates and false-alarm rates, which varies from 0 (no discrimination between studied and unstudied pictures) to 1 (perfect discrimination). Retention interval  = 4 hours.

The results with *Pr*, which combines hits and false alarms into a single measure, shows more clearly the differential impact of the 3-Back task on memory across picture groups. A 3 (Picture Type) ×2 (Secondary Task) ANOVA on *Pr* revealed main effects of picture type [*F*(2,72)  = 83.10, *P<*.001, 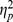  = .70] and Secondary Task [*F*(1,36)  = 14.08, *P<*.001, 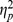  = .28]. Pairwise contrasts yielded significant differences between High and Medium intensity pictures (*M_Pr_*  = .77,.58, *SE*s  = .03,.03, respectively) and between Medium and Low intensity pictures (*M_Pr_*  = .49, *SE*  = .03; *Ps <*.001). In addition, *Pr* was also higher in the 0-Back than in the 3-Back condition (*M_Pr_*  = .70,.53, *SE*s  = .03, *P = *.001). More importantly, the Picture Type × Secondary Task interaction was significant [*F*(2,72)  = 6.75, *P = *.002, 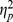  = .16]. Planned contrasts revealed that the difference in *Pr* between N-Back tasks was significant for Medium and Low intensity pictures (*t*s>3.91, *Ps <*.001, Cohen's *d*s*>*1.26, large effect), but not for High intensity pictures (*t* = 1.63, *P = *.11, *d* = 0.53, medium effect). The *Pr* data thus show that cognitive depletion had a clear detrimental effect on memory for neutral and negative pictures with moderate levels of emotional intensity, whereas this effect was cancelled for negative pictures with high levels of emotional intensity. Figure 1 illustrates these results.

#### Recognition data: Remember-Know judgments

Table 3 describes “Remember” and “Know” data. Correct and incorrect R responses (R-HR and R-FAR, respectively) were analysed with two 3 (Picture Type: High, Medium, Low) ×2 (Secondary Task: 0-Back, 3-Back) mixed-design ANOVAs. The first ANOVA revealed main effects of Picture Type [*F*(2,72)  = 94.38, *P<*.001, 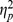  = .72] and Secondary Task [*F*(1,36)  = 16.19, *P<*.001, 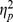  = .31]. Pairwise contrasts showed that R hit rates were higher for High (*M_R-HR_*  = .70, *SE*  = .03) than for Medium intensity pictures (*M_R-HR_*  = .50, *SE*  = .03) and higher for Medium than for Low intensity pictures (*M_R-HR_*  = .37, *SE*  = .03, *Ps <*.001). R hit rates were also higher in the 0-Back condition (*M_R-HR_*  = .62, *SE*  = .04) than in the 3-Back condition (*M_R-HR_*  = .42, *SE*  = .04). The Picture × Task interaction was marginally significant [*F*(2,72)  =  2.70, *P = *.07, 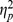  = .07], tentatively suggesting that the increase in cognitive load from the 0-Back to the 3-Back task reduced R hit rates more strongly for Low than for High and Medium intensity pictures. Because the trend was nearly significant and because we had specific hypotheses concerning the effect of cognitive depletion on memory, we further explored this interaction with separate independent-sample *t*-tests for each picture type. The tests revealed that the difference in R-HR between the N-Back tasks was indeed greater for Low (*t* = 4.97, *P<*.001, Cohen's *d* = 1.61) than for High and Medium intensity pictures (*t*s <2.94, *Ps <*.01, *d*s *<*0.95). The ANOVA on incorrect “Remember” responses (R-FAR) yielded no significant main effects or interactions (*F*s <2.5, *P*s>.09).

**Table 3:** Proportion of “Remember” and “Know” responses in recognition memory test as a function of Picture Status, Picture Type and Secondary Task in Experiment 1.

		0-Back					
Picture Status	Picture Type	“Remember”		“Know”		“Old”	
		*M*	*SD*	*M*	*SD*	*M*	*SD*
Old	High	.78	.16	.11	.12	.89	.09
	Medium	.58	.23	.22	.15	.80	.14
	Low	.50	.21	.22	.13	.72	.17
New	High	.01	.02	.07	.10	.08	.11
	Medium	.02	.03	.09	.10	.11	.11
	Low	.02	.03	.10	.08	.12	.08
		3-Back					
Old	High	.61	.19	.22	.15	.83	.08
	Medium	.41	.12	.23	.09	.64	.10
	Low	.24	.08	.30	.11	.54	.10
New	High	.02	.03	.09	.09	.11	.10
	Medium	.03	.06	.14	.12	.17	.13
	Low	.02	.03	.15	.11	.17	.12

*Note.* Proportion of “Old” responses is the sum of the proportion of “Remember” and “Know” responses. The data in the “Know” column correspond to uncorrected “Know” responses. Data for corrected “Know” responses [*K_cor_  =  K/*(1*–R*)] is described in the text. High  =  High emotional intensity; Medium  =  Medium emotional intensity; Low  =  Low emotional intensity (neutral).

Correct and incorrect K responses (K-HR and K-FAR, respectively) were analysed in a similar manner. The proportions of “Know” responses were corrected to take into account the number of “Remember” responses (*K_cor_*). The ANOVA on K_cor_-HR revealed a main effect of Picture Type [*F*(2,72)  = 5.30, *P<*.01, 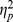  = .13] but no effect of Secondary Task (*F*<1, *P = *.87). Pairwise comparisons showed that K_cor_ hit rates were higher for High (*M_K-HR_*  = .50, *SE*  = .04) than for Low intensity pictures (*M_K-HR_*  = .44, *SE*  = .04, *P*<.01) and Medium intensity pictures (*M_K-HR_*  = .14, *SE*  = .03, *P = *.05). K_cor_ hit rates did not differ between Medium and Low intensity pictures (*P = *.22). The ANOVA on K_cor_-HR also revealed a significant Picture × Task interaction [*F*(2,72)  = 6.51, *P<*.01, 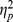  = .15]. However, planned contrasts between N-Back tasks for each picture type did not reveal significant effects.

The ANOVA on K_cor_-FAR revealed only a main effect of Picture Type [*F*(2,72)  = 5.31, *P<*.01, 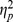  = .13], with lower K_cor_-FAR for High (*M_K-FAR_*  = .08, *SE*  = .02) than for Medium (*M_K-FAR_*  = .12, *SE*  = .02) and Low intensity pictures (*M_K-FAR_*  = .12, *SE*  = .02, *P*s ≤.01). There was no main effect of Secondary Task and no interaction (*F*s <1.7, *P*s>.20).

#### Stimulus-level ANCOVA

Cognitive depletion was estimated for each picture by a difference score (*Pr_0-Back_* – *Pr_3-Back_*). To obtain this difference score, *Pr* values were first calculated for each picture both in the 0-Back condition (*Pr_0-Back_*  =  *HR_0-Back_ – FAR_0-Back_*) and in the 3-Back condition (*Pr_3-Back_  =  HR_3-Back_ – FAR_3-Back_*). These *Pr* values were then subtracted from one another (*Pr_0-Back_ – Pr_3-Back_*), and this value was used as the dependent variable. A large positive *Pr* difference score reflects a strong the impact of cognitive depletion on memory. An ANCOVA on this dependent variable with low-level picture properties as covariates (brightness, contrast, and spatial frequency) was run using Picture Type as the independent variable (High, Medium, Low). The advantage of this analysis is that it allows direct comparisons between high and low emotional intensity conditions while controlling for variations in individual picture properties. The ANCOVA revealed a main effect of Picture Type, *F*(2, 234)  = 6.63, *P<*.01, 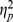  = 05. Independent-sample *t*-tests confirmed that the impairment in memory owing to the 3-Back task was significantly smaller for High (*M_High_*  = .09, *SE*  = .03) than for Low (*M_Low_*  = .23, *SE*  = .02) and Medium intensity pictures (*M_Medium_*  = .23, *SE*  = .03, *P*s <.01), which did not differ from each other (*P* = .99).

These results indicate that emotional intensity reduces the impairment in recognition performance in the 3-Back compared to the 0-back condition. In other words, stimuli with high levels of emotional intensity were more resilient than less emotionally intense stimuli against the memory decrement generated by the secondary task.

#### Secondary task performance

Performance accuracy on the secondary tasks is described in Table 4. A 3 (Picture Type: High, Medium, Low) ×2 (Secondary Task: 0-Back, 3-Back) mixed-design ANOVA on task accuracy revealed main effects of Picture Type [*F*(2,76)  = 3.54, *P* = .03, 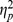  = .09] and Secondary Task [*F*(1,38)  = 30.67, *P<*.001, 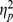  = .45]. Pairwise contrasts showed that accuracy was lower after the presentation of High intensity pictures (*M_acc_*  = .91, *SE*  = .02) than after the presentation of Medium intensity pictures (*M_acc_*  = .94, *SE*  = .01, *P = *.03). However, the High-Low contrast was not significant (Low: *M_acc_*  =  .93, *SE*  = .01, *P = *.09). Accuracy was also higher in the 0-Back condition (*M_0-Back_*  = .99, *SE*  = .02) than in the 3-Back condition (*M_3-Back_*  = .86, *SE*  = .02). The interaction was not significant (*F*<1, *P = *.48).

**Table 4:** Proportion of correct responses (Accuracy) and mean response times (ms) during the study phase in Experiment 1 as a function of Secondary Tasks and Picture Type.

Accuracy	Picture Type					
Task	High		Medium		Low	
	*M*	*SD*	*M*	*SD*	*M*	*SD*
0-Back	.98	.03	.99	.01	.99	.01
3-Back	.84	.13	.87	.10	.86	.11

Response time	Picture Type					
Task	High		Medium		Low	
	*M*	*SD*	*M*	*SD*	*M*	*SD*
0-Back	590	181	584	180	578	157
3-Back	1360	340	1295	295	1282	310

*Notes. 0-Back task*: Participants pressed a button if the number “5” was displayed after each picture presentation. *3-Back task*: Participants pressed a button if the number displayed after each picture presentation was identical to the number displayed three trials before. High  =  High emotional intensity; Medium  =  Medium emotional intensity; Low  =  Low emotional intensity (neutral).

The ANOVA conducted on response times (RTs) revealed a main effect of Secondary Task [*F*(1,38)  = 85.39, *P<*.001, 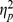  =  .69], reflecting longer RTs to 3-Back (*M_RT_*  = 1312 ms, *SE*  = 56) than to 0-Back trials (*M_RT_*  = 584 ms, *SE*  = 56). There was also a main effect of Picture Type [*F*(2,76)  = 5.32, *P<*.01, 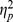  = .12] and a Type × Task interaction [*F*(2,76)  = 3.13, *P = *.049, 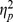  = .08]. This interaction was driven by a significant effect of Picture Type only in the 3-Back condition [*F*(2,38)  = 4.64, *P = *.02, 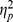  = .20], with longer response times following High intensity pictures compared to Medium and Low intensity pictures (*M_RT_*  = 1360, 1295, 1282 ms, *SE*  = 76, 66, 69, *Ps <.*05). Response times between Medium and Low intensity pictures did not differ significantly.

## Experiment 2

One of the key results of Experiment 1 is that negative pictures with moderate intensity levels (the "medium" intensity stimuli) provided little memory protection against cognitive depletion. When cognitive load was increased at encoding, recognition of medium- and low-intensity pictures decreased in a similar manner. By contrast, recognition of high-intensity negative pictures was barely affected. These results indicate that moderately negative stimuli are not protected against working-memory-related disruption. It is possible, however, that the relatively short study-test interval used in Experiment 1 (4 hours) was not long enough to allow for a full development of the memory protection afforded by moderately negative stimuli. This would be consistent with evidence showing a positive relationship between study-test interval and emotion-enhanced memory performance [31], [32].

Therefore, Experiment 2 had two goals: First, we examined whether the results of Experiment 2 could be replicated on a different sample. Second, we examined if the lower memory protection associated with medium-intensity pictures would also be observed when the study-test delay was longer (2 days). If the difference between high- and medium-intensity pictures in Experiment 1 was caused by its relatively short study-test interval, then increasing this interval in Experiment 2 should allow medium-intensity pictures to achieve stronger levels of memory protection when compared to low-intensity (neutral) pictures.

### Method

#### Participants

Forty adults (*M_age_*  = 19.7, *SD*  = 1.5; 7 males) took part in this experiment. Participants were either paid £10 or rewarded with course credits and were randomly assigned to one of the N-Back conditions. Two participants from the 0-Back condition were outliers (hit rates beyond 2.5 *SD*s from their group average) and were excluded from the recognition data. All participants signed an informed consent and the study was approved by the Ethics Committee of the Department of Psychology at Durham University.

#### Stimuli and Design

We used a total of 320 images (160 negative and 160 neutral) rated in a similar manner as the images used in Experiment 1. As in Experiment 1, images were taken from IAPS and “Google Images”. They were split into two lists of 160 images (80 negative and 80 neutral). These lists were used as *Old* or *New* stimuli and were counterbalanced across participants. Negative pictures were further classified as high intensity (*High*) and low intensity pictures (*Medium*) using a median split on their arousal ratings. As in Experiment 1, neutral pictures formed a low-intensity picture set (*Low*). The three picture groups (High, Medium, Low) were significantly different in valence [*F*(2,317)  = 846.38, *P<*.001, 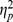  = .84] and arousal [*F*(2,317)  = 711.57, *P<*.001, 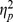  = .82]. As in Experiment 1, picture groups were matched for content (similar number of pictures featuring humans, faces, animals and objects) and for low-level visual features (similar average levels of brightness, contrast, and spatial frequency across groups; *Ps>*.08). Picture properties are summarized in Table 5.

**Table 5:** Stimulus properties by Picture Type in Experiment 2.

	Picture Type					
	High		Medium		Low	
Property	*M*	*SD*	*M*	*SD*	*M*	*SD*
Valence	1.57	0.33	2.25	0.26	3.21	0.30
Arousal	3.83	0.33	2.85	0.30	2.03	0.38
Brightness	108.89	30.67	98.22	33.78	100.95	30.77
Contrast	12.93	6.10	15.13	7.08	13.41	6.60
Spatial freq.	17.76	10.94	17.86	11.25	18.32	10.79

*Note.* High  =  High emotional intensity; Medium  =  Medium emotional intensity; Low  =  Low emotional intensity (neutral).

#### Procedure

The experimental procedure was the same as in Experiment 1, except that the study-test interval was 2 days instead of 4 hours.

#### Data analysis

Data analysis was the same as in Experiment 1.

### Results

#### Recognition data: Hits, false alarms and Pr

A 3 (Picture Type: High, Medium, Low) ×2 (Secondary Task: 0-Back, 3-Back) mixed-design ANOVA on hit rates revealed main effects of Picture Type [*F*(2,72)  = 220.34, *P<*.001, 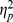  = .86] and Secondary Task [*F*(1,36)  = 29.23, *P<*.001, 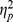  = .45]. Pairwise comparisons showed significant differences between High and Medium intensity pictures (*M_HR_*  = .82,.58, *SE*s  = .02,.02, respectively) and between Medium and Low pictures (*M_HR_*  = .45, *SE*  = .02; *Ps <*.001). Hit rates were higher in the 0-Back than in the 3-back condition (*M_HR_*  = .70,.53, *SE*s  = .02). More importantly, the interaction was highly significant [*F*(2,72)  = 10.62, *P<*.001, 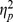  = .23]. Planned contrasts confirmed that the difference in HR between 0- and 3-Back tasks was greater for Low and Medium intensity pictures (*t*s>5.5, *Ps <*.001, *d*s>1.91, large effect) than for High intensity pictures (*t* = 2.3, *P* = .03, *d* = 0.74, medium effect).

For false-alarm data, there was only a marginal effect of Picture Type [*F*(2,72)  = 2.52, *p* = .09, 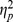  = .07, driven by slightly higher errors to High than to Medium and Low pictures (*M_FAR_*  =  .18,.14,.15, *SE*s  = .02). Table 6 summarises these results.

**Table 6:** Proportion of “Old” responses in the recognition memory test as a function of Picture Type, Picture Status and Secondary Task (Experiment 2).

		Picture Type					
N-Back task	Picture status	High		Medium		Low	
		*M*	*SD*	*M*	*SD*	*M*	*SD*
0-Back	Old	.86	.08	.69	.12	.57	.11
	New	.19	.12	.15	.09	.18	.10
3-Back	Old	.78	.13	.47	.11	.34	.14
	New	.17	.16	.13	.10	.12	.09

*Note. Hit rate*  =  Proportion of “Old” responses to *Old* pictures; *False-alarm rate*  =  Proportion of “Old” responses to *New* pictures. High  =  High emotional intensity; Medium  =  Medium emotional intensity; Low  =  Low emotional intensity (neutral).

**Figure 2:**
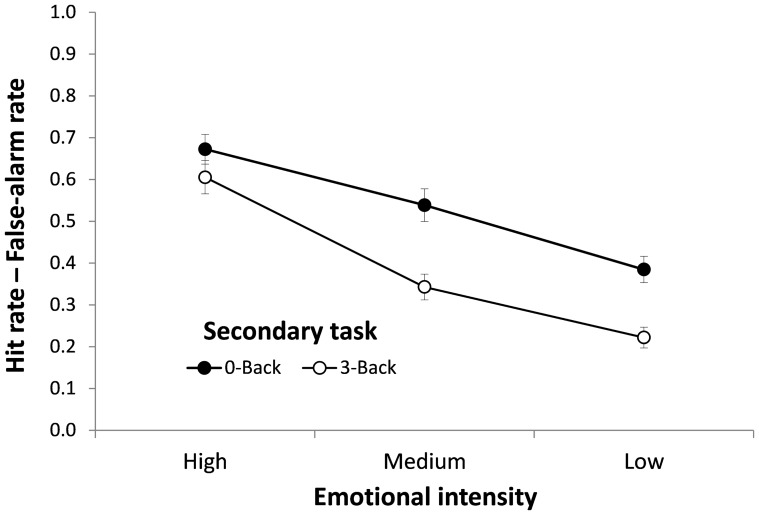
Recognition memory as a function of Picture Type and Secondary Task (Experiment 2). Memory was measured with *Pr* (the difference between hit rates and false-alarm rates). Retention interval  = 2 days.

The results with *Pr* confirm the pattern described above. A 3 (Picture Type) ×2 (Secondary Task) ANOVA on *Pr* revealed main effects of Type [*F*(2,72)  = 153.55, *P<*.001, 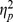  = .81] and Task [*F*(1,36)  = 11.07, *P* = .002, 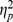  = .24]. Pairwise contrasts yielded significant differences between High and Medium intensity pictures (*M_Pr_*  = .64,.44, *SE*s  = .03,.03, respectively) and between Medium and Low intensity pictures (*M_Pr_*  = .31, *SE*  = .02; *Ps <*.001). *Pr* was also higher in the 0-Back than in the 3-Back condition (*M_Pr_*  = .53,.39, *SE*s  = .03). Similar to Experiment 1, the Type × Task interaction was significant [*F*(2,72)  = 6.16, *P* = .002, 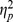  = .15]. Planned contrasts revealed that the difference in *Pr* between N-Back conditions was significant for Medium and Low intensity pictures (*t*s>3.96, *Ps <*.001, *d*s*>*1.28, large effect), but not for High intensity pictures (*t* = 1.23, *p* = .23, *d* = 0.36, small effect). These results fully replicate Experiment 1: *Pr* data in Experiment 2 confirmed that cognitive depletion had a larger detrimental effect on memory for neutral and negative pictures with moderate levels of emotional intensity than for high-intensity negative pictures. Figure 2 depicts these results.

#### Recognition data: Remember-Know judgments

Table 7 describes “Remember” and “Know” data. Correct and incorrect R responses (R-HR and R-FAR, respectively) were analysed with two 3 (Picture Type) ×2 (Secondary Task) mixed-design ANOVAs. The first ANOVA revealed main effects of Type [*F*(2,72)  = 71.73, *P<*.001, 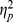  = .67] and Task [*F*(1,36)  = 5.96, *P = *.02, 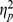  = .14]. Pairwise contrasts showed that R hit rates were higher for High (*M_R-HR_*  = .50, *SE*  = .03) than for Medium intensity pictures (*M_R-HR_*  = .31, *SE*  = .03) and higher for Medium than for Low intensity pictures (*M_R-HR_*  = .19, *SE*  = .02, *Ps <*.001). R hit rates were also higher in the 0-Back condition (*M_R-HR_*  =  .39, *SE*  = .03) than in the 3-Back condition (*M_R-HR_*  =  .28, *SE*  = .03). The Type × Task interaction was not significant [*F*<1, *P = *.56].

**Table 7:** Proportion of “Remember” and “Know” responses in recognition memory test as a function of Picture Status, Picture Type and Secondary Task in Experiment 2.

		0-Back					
Picture Status	Picture Type	“Remember”		“Know”		“Old”	
		*M*	*SD*	*M*	*SD*	*M*	*SD*
Old	High	.54	.21	.33	.19	.86	.08
	Medium	.38	.19	.31	.13	.69	.12
	Low	.24	.15	.35	.13	.57	.11
New	High	.04	.06	.14	.11	.19	.12
	Medium	.04	.05	.12	.07	.15	.09
	Low	.03	.05	.14	.10	.18	.10
		3-Back					
Old	High	.45	.20	.33	.19	.78	.13
	Medium	.24	.13	.24	.14	.47	.11
	Low	.14	.10	.21	.10	.34	.14
New	High	.05	.09	.13	.14	.17	.16
	Medium	.03	.05	.10	.09	.13	.10
	Low	.03	.06	.19	.15	.12	.09

*Note.* Proportion of “Old” responses is the sum of the proportion of “Remember” and “Know” responses. The data in the “Know” column correspond to uncorrected “Know” responses. Data for corrected “Know” responses [*K_cor_  =  K/*(1*–R*)] is described in the text. High  =  High emotional intensity; Medium  =  Medium emotional intensity; Low  =  Low emotional intensity (neutral).

The ANOVA on incorrect “Remember” responses (R-FAR) yielded a main effect of Picture Type [*F*(2,72)  = 4.52, *P = *.01, 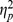  = .11], reflecting a higher R-FAR for High (*M_R-FAR_*  = .05, *SE*  =  .01) than for Medium (*M_R-FAR_*  = .03, *SE*  = .01, *P = *.04) and Low intensity pictures (*M_R-FAR_*  = .02, *SE*  = .01, *P = *.03). There was, however, no effect of Secondary Task and no interaction (*F*s <1, *P*s>.73).

The ANOVA on K_cor_-HR revealed main effects of Picture Type [*F*(2,72)  = 62.41, *P<*.001, 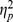  = .63] and Secondary Task [*F*(1,36)  = 17.93, *P<*.001, 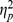  = .33]. Pairwise comparisons showed that K_cor_ hit rates were higher for High (*M_K-HR_*  = .64, *SE*  = .03) than for Medium intensity pictures (*M_K-HR_*  = .41, *SE*  =  .03, *P*<.001) and that K_cor_ hit rates were higher for Medium than for Low intensity pictures (*M_K-HR_*  = .36, *SE*  = .02, P = .06). The Type × Task interaction was not significant (*F* = 1.64, *P = *.20]. The ANOVA on K_cor_-FAR yielded no significant effect (*F*s <1.7, *P*s>.17). These results indicate that the effects of protection against cognitive depletion are not reflected in judgments of "remembering" or "knowing". instead, they are observed in objective recognition parameters (Hit rate and *Pr*).

#### Stimulus-level ANCOVA

The same ANCOVA used for Experiment 1 was run on Experiment 2 data, leading to similar results. Unsurprisingly, the ANCOVA revealed a main effect of Picture Type, *F*(2, 314)  = 5.06, *P<*.01, 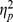  = 03. *Post-hoc t*-tests confirmed that recognition performance for High intensity pictures (*M_High_*  = .06, *SE*  = .03) was less disrupted by the 3-Back task than performance for Low (*M_Low_*  = .17, *SE*  = .02) and Medium intensity pictures (*M_Medium_*  = .17, *SE*  = .03, *P<*.01). No difference was found between Medium and Low intensity pictures (*P* = .83).

Experiment 2 replicated the results of Experiment 1. Negative stimuli with high intensity levels were more protected against cognitive depletion than neutral stimuli and negative stimuli with moderate intensity levels. This pattern of results was found when using both standard analyses (ANOVAs on *Pr*) and stimulus-level analyses. In particular, we observed that the protective effects of emotion against cognitive depletion remained different between high- and medium-intensity pictures even after a 2-day retention interval, suggesting that the relatively short interval in Experiment 1 was probably not the main factor behind this effect. This effect was confirmed by cross-experiment analyses reported in the supplementary section (File S1).

#### Secondary task performance

Performance on the secondary tasks is described in Table 8. A 3 (Picture Type:) ×2 (Secondary Task) ANOVA on task accuracy revealed only a main effect of Task [*F*(1,38)  = 49.07, *P<*.001, 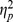  = .56]: accuracy was also higher in the 0-Back condition (*M_0-Back_*  = .99, *SE*  = .01) than in the 3-Back condition (*M_3-Back_*  = .85, *SE*  = .01). The main effect of Picture Type and the interaction term were not significant (*F*s <1, *P*s>.39). As with accuracy, the ANOVA on mean response times revealed only a main effect of Task [*F*(1,38)  = 48.92, *P<*.001, 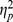  = .56], reflecting longer RTs to 3-back (*M_RT_*  =  1495 ms, *SE*  = 93) than to 0-back trials (*M_RT_*  = 570 ms, *SE*  = 93). There was no effect of Picture Type and no Picture type × Task interaction (*F*s <1.13, *P*s*>*.32).

**Table 8:** Proportion of correct responses (Accuracy) and mean response times (ms) during the study phase in Experiment 2 as a function of Secondary Tasks and Picture Type.

Accuracy	Picture Type					
Task	High		Medium		Low	
	*M*	*SD*	*M*	*SD*	*M*	*SD*
0-Back	.99	.01	.99	.01	.99	.02
3-Back	.84	.11	.84	.10	.86	.10

Response time	Picture Type					
Task	High		Medium		Low	
	*M*	*SD*	*M*	*SD*	*M*	*SD*
0-Back	579	98	564	90	559	86
3-Back	1505	569	1473	583	1507	615

*Notes. 0-Back task*: Participants pressed a button if the number “5” was displayed after each picture presentation. *3-Back task*: Participants pressed a button if the number displayed after each picture presentation was identical to the number displayed three trials before. High  =  High emotional intensity; Medium  =  Medium emotional intensity; Low  =  Low emotional intensity (neutral).

## General Discussion

In two studies, we found that negative emotional content can reduce and even cancel the impact of cognitive depletion on recognition memory. Crucially, this study is the first to formally demonstrate that this effect is specific to items with high levels of emotional intensity, as emotional items with moderate or low intensity levels were clearly affected by cognitive depletion. These results are consistent with recall studies that have previously demonstrated smaller divided-attention memory costs for emotional information [3], [4]; and they extend previous research showing that this phenomenon can also be observed in recognition tasks if highly intense emotional stimuli are used. These results were replicated in two different samples, using different study-test intervals (4 hours and 2 days). In addition, covariate analyses established that our results cannot be explained by low-level picture properties thought to correlate with picture emotionality [26].

Our results contrast with previous research in which no evidence of an emotional reduction of cognitive depletion effects was apparent [5], [6]. Two aspects of our experimental methods may explain this discrepancy. First, we took into account varying levels of intensity within our emotional stimuli. As explained in the Introduction, there is ample evidence that high-intensity emotional stimuli can have effects on memory that can be distinguished from low-intensity emotional stimuli [10], [11], [24], [33], [34]. Previous studies that used simple “emotional vs. neutral” contrasts may have masked any protective effects from high-intensity emotional contents, because these were merged with lower intensity stimuli. Second, the study-test intervals used here were longer than those in most previous studies. Longer intervals, from hours to days, enhance the memory advantage of emotional stimuli in recognition tasks [13], [14]. Adopting a methodological standard of longer retention intervals may thus have helped to detect the protective role of emotion against the effects of divided attention. However, to fully investigate this possibility, future research will need to contrast long retention intervals with conditions where memory tests are performed immediately after encoding.

How does emotion protect memory against cognitive depletion at encoding? A potential account involves the special attention-grabbing properties of emotional stimuli. High-arousal negative stimuli are known to mobilize attentional resources to a greater extent than low-arousal stimuli [35]–[41], and overt attention plays an important role in the emotional enhancement of memory [4], [23]. If more attentional resources remained available to high-intensity compared to medium- and low-intensity stimuli while participants performed the 3-Back task, then high-intensity stimuli would benefit from better encoding and, consequently, better recognition than medium- and low-intensity stimuli. In other words, the additional attentional resources captured by high-intensity stimuli would compensate for the attentional deficit caused by the secondary 3-Back task. This account is consistent with classical models of emotion-memory interactions [42]–[44] and more recent accounts which emphasize the notion that attention can mediate the emotional enhancement of memory [23], [45]. These models suggest that, in the context of emotional memory, emotional items might attract cognitive resources due to their intrinsic motivational relevance [46], or because emotional items are more distinctive when presented alongside neutral items [47], or because emotional items are more interrelated than neutral items and therefore provide more opportunities for organizational encoding strategies [48].

Consistent with this account, we found in Experiment 1 a Picture Type × Secondary task interaction in response times. The interaction was driven by longer response times to 3-Back trials following high-intensity pictures. This finding may suggest that high-intensity pictures took away attentional resources needed for the secondary task, which therefore became more difficult. However, this attentional account cannot explain all our results. The same Picture Type × Secondary Task interaction in RT was not significant in Experiment 2, nor was it significant when looking at secondary task accuracy. In addition, models emphasizing that emotional effects on memory are mediated by attention and other related cognitive properties (distinctiveness and relatedness) are thought to be mainly applicable when memory tests follow immediately the encoding stage [49]. Emotional effects on memory paradigms using longer study-test delays, such as the current studies, may also be governed by neuromodulatory mechanisms (as explained later in this discussion), which could potentially mask the effects of attentional processes related to the encoding stage. It has to be noted that, although the absence of interaction in the N-back data of Experiment 2 suggests that overt attention is not a reliable explanation for our findings, it does not exclude a potential contribution of automatic forms of attention that would not be affected by our 3-Back manipulation [9], [23]

Alternatively, the vividness and fluency associated with the encoding of high-intensity pictures could partially explain their immunity against the effects of divided attention on memory. High-arousal information is encoded more easily [50] and leads to more vivid memories [51], [52] than low-arousal stimuli. More specifically, recognition of these stimuli is often associated with higher levels of memory vividness, measured with “Remember” responses [10], [33], [53]. The results from Experiments 1 and 2 showed that high-intensity pictures yielded more correct “Remember” responses than medium- and low-intensity pictures, suggesting that high-intensity pictures were encoded more vividly than medium-intensity pictures. However, an account based purely on vividness cannot fully explain our results, as the Picture Type × Secondary Task interaction for correct “Remember” responses was only marginally significant in Experiment 1 and non-significant in Experiment 2. More specifically, the difference in “Remember” responses between high- and medium-intensity pictures was not significantly stronger in the 3-Back condition relative to the 0-Back condition, a result that would indicate a relative sparing of the effects of divided attention on the vividness of high-intensity pictures. Thus, the data are not consistent with the view that vividness was the main factor contributing to memory protection against cognitive depletion.

A more plausible explanation may be derived from theoretical models of emotional memory which emphasize the modulatory role that several emotion-related neurotransmitters and hormones have on memory systems [15], [54]–[56]. These models suggest that neurotransmitters and hormones released during an emotional situation can strengthen the formation and consolidation of memory traces, making them particularly resistant to decay [57]. This mechanism is mediated by the amygdala, and it takes place over a period of time that extends well after the encoding stage [15]. These models are very useful to explain the effects of emotion on recognition memory, because these effects are usually stronger after a long study-test interval [31], [32]. In addition, these models are useful to explain parametric effects of emotional intensity on memory (i.e., the effects of graded levels of emotional intensity on memory [10], [11], [24]). Applied to the present study, it could be speculated that cognitive interference disrupts encoding by weakening the creation of a memory trace, making it more vulnerable to temporal decay. However, the neuromodulation triggered by arousing contents of high-intensity pictures could potentially counteract the effects of temporal decay by strengthening the trace through long-term consolidation. The results presented here are consistent with neuromodulatory models of emotional memory effects in that (*i*) the effects were obtained after a long study-test interval during which the consolidation processes described in these models could have taken place; (*ii*) the effects were dependent on a graded manipulation of emotional intensity, in which only the highest level of emotional intensity provided a protection against cognitive depletion. This is consistent with evidence suggesting that high levels of emotional intensity are linked to neuromodulatory mechanisms that are distinct from moderate and low levels of emotional intensity [10], [24], [55], [58].

The results reported in this article raise further questions that need to be discussed. First, the concept of “emotional intensity” has significant overlaps with the concept of “arousal”. Emotional intensity is a concept that has often been used in the literature of emotional processes and usually refers to the perceived strength of an emotional reaction to a stimulus or a situation [52], [59], [60]. The concept of arousal is often used in conjunction with emotional pictures or words, and most of the time, refers to the self-reported strength of an emotional state triggered by one of these stimuli [60], [61]. The concept of arousal is often used in contrast to the concept of valence, which refers to a bipolar “positive vs. negative” dimension, and arousal is often seen as independent from valence. However, when valence is unipolar as in the present study (neutral vs. negative), valence and arousal tend to correlate and probably refer to an underlying common unipolar perception of the strength of emotional activation. To explore this question, we performed multiple regression analyses that explored if the effects of unipolar valence and arousal could be disentangled. Although the results of Experiment 1 suggest that arousal has a unique moderating effect on cognitive depletion, the same analyses were inconclusive for Experiment 2 (see File S1), in which the effects of arousal and valence could not be disentangled. This finding argues in favour of the thesis that unipolar valence and arousal cannot be reliably distinguished in this context, and that the findings reported here are better accounted for by an underlying factor of negative emotional intensity. Future research will be needed to establish if the effects reported in this article can be accounted for by a dimension of arousal that is independent from a *bipolar* dimension of valence.

Second, as explained above, although emotional intensity was the main driver of the effects reported above, it is important to note that only neutral and negative stimuli, but not positive stimuli, were used in this study. It is possible that positive stimuli respond differently to cognitive depletion. In fact, neuroimaging studies have shown that the valence of a subsequently-remembered stimulus affects which neural systems are engaged at encoding: negative stimuli preferentially recruit brain regions associated with sensory processing, whereas positive stimuli preferentially recruit regions linked to semantic processing [62], [63]. To the extent that negative and positive stimuli engage distinct neural processes at encoding, they could also be differently affected by a secondary task. However, data from attention and memory studies indicate that arousing stimuli can behave in similar ways regardless of valence [64]. For instance, arousal equally improves memory for the spatial location of negative and positive stimuli in both recall and recognition tasks [65]. Moreover, negative and positive stimuli with similar levels of arousal are equally affected by cognitive depletion at encoding [[66], Exp. 3]. These results show that, despite engaging different neural processes at encoding, positive and negative stimuli may behave in a similar manner in standard behavioural tasks. Further research will be necessary to assess the impact of cognitive depletion on memory as a function of bipolar valence. In particular, these studies could address the putative encoding differences between negative and positive stimuli with tasks that tap more specifically sensory and semantic processes. For example, cognitive depletion at encoding could have different mnemonic consequences to negative and positive stimuli if the secondary tasks require perceptual or semantic judgements. Likewise, potential differences in valence could be uncovered at retrieval by contrasting performance in yes-no recognition tasks, which tend to engage more semantic, gist-based processes, and source recognition tasks, which engage more perceptual, recollection-based processes [67]–[69].

Third, it could be argued that we could have performed mediation analyses similar to our previous work [23] to assess the role of attention in the effects of emotion on memory performance. Although this was not the goal of the studies presented in the current paper, we acknowledge that it would have been an interesting secondary question to address. However, mediation analyses were not performed in this study for two reasons. First, mediation analyses speak more directly to models which posit that cognitive factors (in particular attentional factors) are sufficient to account for immediate memory effects [23], [45]. In our design, however, retention intervals were long enough to allow neuromodulatory mechanisms to influence performance [15], above and beyond potential effects of attentional changes at encoding. Thus, mediation analyses here would be unlikely to provide a process-pure test of attentional accounts of emotional memory effects. In addition, the secondary task used in this study did not provide a trial-by-trial measure of working memory that would be amenable to mediation analyses. Although participants entered secondary task responses on a trial-by-trial basis, the temporally extended nature of the N-Back task (which relies on information stored *N* trials earlier) clouds the interpretation of cognitive costs specific to a single trial. This feature of N-Back tasks prevents the utilization of mediation analyses to test attentional models of emotional memory effects, because trial-specific cognitive costs are one of the main units of analysis necessary for a mediation-based approach of this question [23], [45]. However, it is important to note that this apparent limitation does not affect the main goal of the current study, which was to examine if the effects of cognitive depletion on memory were affected by emotional intensity. In addition, the choice of N-Back as our secondary task was motivated by previous research showing that the task can (*i*) reliably interfere with emotional memory over long intervals [70]; (*ii*) impose significant demands on cognitive resources [18]; (*iii*) provide a manipulation of divided attention while controlling for viewing conditions [20].

Fourth, it could be argued that the 0-Back task could also recruit cognitive control resources as it can be assimilated to a task-switching context in which the encoding task alternates with the digit task. Although this possibility cannot be fully excluded, the behavioural results demonstrate that the 3-Back task imposed a much heavier burden on cognitive resources than the 0-Back task. Therefore, if any effects related to cognitive demands imposed by the 0-back task occurred, they seem to have been significantly outweighed by the demands imposed by the 3-back condition. Finally, the secondary task used in the current studies differs from most previous studies that typically involved requiring participants to perform an actual attentional task at the same time of encoding. In the 3-Back condition of the present studies, participants were simply led to use working memory processes during encoding. Therefore, one could argue that the depletion implemented in the present studies relies on working memory processes, which could explain the differences between our data and previous research. We acknowledge this possibility, which could suggest that the depletion-protective effect of emotion depends on the specific type of depletion being implemented. However, it has to be said that working memory and attention are often thought to be inherently linked [71], [72] and it is not excluded that working memory processes may have been necessary to perform the distracting attentional tasks used in previous studies [23].

In conclusion, we showed across two studies that recognition memory for stimuli with high levels of negative emotional intensity is protected against the effects of cognitive depletion at encoding. Recognition memory was impaired when a secondary 3-Back working-memory task was performed at encoding, but only when the stimuli consisted of pictures with medium or low emotional intensity. When pictures had high levels of emotional intensity, cognitive depletion did not impair recognition memory. These results extend previous research by showing that, under certain conditions, encoding of emotional stimuli can become relatively immune to cognitive depletion.

## Supporting Information

File S1 **Supplementary analyses.** Further analyses were carried out to explore the data on confidence ratings and to assess the separate effects of valence, arousal, and study-test interval on memory performance.
(DOCX)

## References

[1] Naveh-Benjamin M, Guez J (2000) Effects of divided attention on encoding and retrieval processes: Assessment of attentional costs and a componential analysis. J Exp Psychol Learn Mem Cogn 26: 1461–1482. 11185777 10.1037//0278-7393.26.6.1461

[2] Hicks JL, Marsh RL (2000) Toward specifying the attentional demands of recognition memory. J Exp Psychol Learn Mem Cogn 26: 1483–1498. 11185778 10.1037//0278-7393.26.6.1483

[3] Kern RP, Libkuman TA, Otani H (2005) Emotional stimuli, divided attention, and memory. Emotion 5: 408–417. 16366745 10.1037/1528-3542.5.4.408

[4] Talmi D, Schimmack U, Paterson T, Moscovitch M (2007) The role of attention and relatedness in emotionally enhanced memory. Emotion 7: 89–102. 17352566 10.1037/1528-3542.7.1.89

[5] Clark-Foos A, Marsh RL (2008) Recognition memory for valenced and arousing materials under conditions of divided attention. Memory 16: 530–537. 18569681 10.1080/09658210802007493

[6] Kensinger EA, Corkin S (2003) Effect of divided attention on the memory benefit for negative as compared to neutral words. Brain Cognition 51: 223–225.

[7] D'Argembeau A, Van der Linden M (2007) Facial expressions of emotion influence memory for facial identity in an automatic way. Emotion 7: 507–515. 17683207 10.1037/1528-3542.7.3.507

[8] Kensinger EA, Corkin S (2004) Two routes to emotional memory: Distinct neural processes for valence and arousal. Proc Natl Acad Sci U S A 101: 3310–3315. 14981255 10.1073/pnas.0306408101 PMC365786

[9] Maddox GB, Naveh-Benjamin M, Old S, Kilb A (2012) The role of attention in the associative binding of emotionally arousing words. Psychon B Rev 19: 1128–1134. 10.3758/s13423-012-0315-x 23055140

[10] Schaefer A, Pottage CL, Rickart AJ (2011) Electrophysiological correlates of remembering emotional pictures. Neuroimage 54: 714–724. 20650320 10.1016/j.neuroimage.2010.07.030

[11] van Stegeren AH, Goekoop R, Everaerd W, Scheltens P, Barkhof F, et al (2005) Noradrenaline mediates amygdala activation in men and women during encoding of emotional material. Neuroimage 24: 898–909. 15652324 10.1016/j.neuroimage.2004.09.011

[12] Sharot T, Yonelinas AP (2008) Differential time-dependent effects of emotion on recollective experience and memory for contextual information. Cognition 106: 538–547. 17451666 10.1016/j.cognition.2007.03.002

[13] Sharot T, Phelps EA (2004) How arousal modulates memory: Disentangling the effects of attention and retention. Cogn Affect Behav Neurosci 4: 294–306. 15535165 10.3758/cabn.4.3.294

[14] Payne JD, Kensinger EA (2010) Sleep's role in the consolidation of emotional episodic memories. Curr Dir Psychol Sci 19: 290–295.

[15] McGaugh JL (2004) The amygdala modulates the consolidation of memories of emotionally arousing experiences. Annu Rev Neurosci 27: 1–28. 15217324 10.1146/annurev.neuro.27.070203.144157

[16] Schaefer A, Braver TS, Reynolds JR, Burgess GC, Yarkoni T, et al (2006) Individual differences in amygdala activity predict response speed during working memory. J Neurosci 26: 10120–10128. 17021168 10.1523/JNEUROSCI.2567-06.2006 PMC1851922

[17] Gray JR, Burgess GC, Schaefer A, Yarkoni T, Larsen RJ, et al (2005) Affective personality differences in neural processing efficiency confirmed using fMRI. Cogn Affect Behav Neurosci 5: 182–190. 16180624 10.3758/cabn.5.2.182

[18] Braver TS, Cohen JD, Nystrom LE, Jonides J, Smith EE, et al (1997) A parametric study of prefrontal cortex involvement in human working memory. Neuroimage 5: 49–62. 9038284 10.1006/nimg.1996.0247

[19] Fales CL, Barch DM, Burgess GC, Schaefer A, Mennin DS, et al (2008) Anxiety and cognitive efficiency: differential modulation of transient and sustained neural activity during a working memory task. Cogn Affect Behav Neurosci 8: 239–253. 18814461 10.3758/cabn.8.3.239

[20] King R, Schaefer A (2011) The emotional startle effect is disrupted by a concurrent working memory task. Psychophysiology 48: 269–276. 20636296 10.1111/j.1469-8986.2010.01062.x

[21] Lang PJ, Bradley MM, Cuthbert BN (2001) International affective picture system (IAPS): Affective ratings of pictures and instruction manual. Gainesville: University of Florida.

[22] Bradley MM, Lang PJ (1994) Measuring emotion: The self-assessment manikin and the semantic differential. J Behav Ther Exp Psy 25: 49–59. 10.1016/0005-7916(94)90063-9 7962581

[23] Pottage CL, Schaefer A (2012) Visual attention and emotional memory: Recall of aversive pictures is partially mediated by concurrent task performance. Emotion 12: 33–38. 22059519 10.1037/a0024574

[24] Schaefer A, Fletcher K, Pottage CL, Alexander K, Brown C (2009) The effects of emotional intensity on ERP correlates of recognition memory. Neuroreport 20: 319–324. 19188857 10.1097/WNR.0b013e3283229b52

[25] Dolcos F, LaBar KS, Cabeza R (2004) Dissociable effects of arousal and valence on prefrontal activity indexing emotional evaluation and subsequent memory: An event-related fMRI study. Neuroimage 23: 64–74. 15325353 10.1016/j.neuroimage.2004.05.015

[26] Bradley MM, Hamby S, Low A, Lang PJ (2007) Brain potentials in perception: Picture complexity and emotional arousal. Psychophysiology 44: 364–373. 17433095 10.1111/j.1469-8986.2007.00520.x

[27] Rajaram S (1993) Remembering and knowing: Two means of access to the personal past. Mem Cognition 21: 89–102. 10.3758/bf03211168 8433652

[28] Gardiner JM, Java RI (1993) Recognition memory and awareness: An experimental approach. Eur J Cogn Psychol 5: 337–346.

[29] Yonelinas AP (2001) Consciousness, control, and confidence: The 3 Cs of recognition memory. J Exp Psychol Gen 130: 361–379. 11561915 10.1037//0096-3445.130.3.361

[30] Song Z, Wixted JT, Hopkins RO, Squire LR (2011) Impaired capacity for familiarity after hippocampal damage. Proc Natl Acad Sci U S A 108: 9655–9660. 21606344 10.1073/pnas.1107247108 PMC3111286

[31] Sharot T, Verfaellie M, Yonelinas AP (2007) How emotion strengthens the recollective experience: a time-dependent hippocampal process. PLoS ONE 2: e1068. 17971848 10.1371/journal.pone.0001068 PMC2031918

[32] Sharot T, Yonelinas AP (2008) Differential time-dependent effects of emotion on recollective experience and memory for contextual information. Cognition 106: 538–547. 17451666 10.1016/j.cognition.2007.03.002

[33] Ochsner KN (2000) Are affective events richly recollected or simply familiar? The experience and process of recognizing feelings past. J Exp Psychol Gen 129: 242–261. 10868336 10.1037//0096-3445.129.2.242

[34] Kensinger EA, Corkin S (2004) Two routes to emotional memory: distinct neural processes for valence and arousal. Proc Natl Acad Sci U S A 101: 3310–3315. 14981255 10.1073/pnas.0306408101 PMC365786

[35] Pessoa L (2008) On the relationship between emotion and cognition. Nat Rev Neurosci 9: 148–158. 18209732 10.1038/nrn2317

[36] Vuilleumier P (2005) How brains beware: Neural mechanisms of emotional attention. Trends Cognit Sci 9: 585–594. 16289871 10.1016/j.tics.2005.10.011

[37] Anderson AK (2005) Affective influences on the attentional dynamics supporting awareness. J Exp Psychol Gen 134: 258–281. 15869349 10.1037/0096-3445.134.2.258

[38] Vuilleumier P, Huang YM (2009) Emotional Attention: Uncovering the Mechanisms of Affective Biases in Perception. Curr Dir Psychol Sci 18: 148–152.

[39] Huang YM, Baddeley A, Young AW (2008) Attentional capture by emotional stimuli is modulated by semantic processing. J Exp Psychol Human 34: 328–339. 10.1037/0096-1523.34.2.328 18377174

[40] Öhman A, Mineka S (2001) Fears, phobias, and preparedness: Toward an evolved module of fear and fear learning. Psychol Rev 108: 483–522. 11488376 10.1037/0033-295x.108.3.483

[41] Mermillod M, Droit-Volet S, Devaux D, Schaefer A, Vermeulen N (2010) Are coarse scales sufficient for fast detection of visual threat? Psychol Sci 21: 1429–1437. 20817781 10.1177/0956797610381503

[42] Christianson SA, Loftus EF, Hoffman H, Loftus GR (1991) Eye fixations and memory for emotional events. J Exp Psychol Learn Mem Cogn 17: 693–701. 1832433 10.1037//0278-7393.17.4.693

[43] Christianson SA (1992) Emotional stress and eyewitness memory: a critical review. Psychol Bull 112: 284–309. 1454896 10.1037/0033-2909.112.2.284

[44] Loftus EF, Loftus GR, Messo J (1987) Some facts about weapon focus. Law Human Behav 11: 55–62.

[45] Talmi D, Schimmack U, Paterson T, Moscovitch M (2007) The role of attention and relatedness in emotionally enhanced memory. Emotion 7: 89–102. 17352566 10.1037/1528-3542.7.1.89

[46] Schmidt SR, Saari B (2007) The emotional memory effect: differential processing or item distinctiveness? Mem Cognit 35: 1905–1916. 10.3758/bf03192924 18265607

[47] Watts S, Buratto LG, Brotherhood EV, Barnacle EV, Schaefer A (2014) The neural fate of neutral information in emotion-enhanced memory. Psychophysiology 51: 673–684. 24673606 10.1111/psyp.12211

[48] Talmi D (2013) Enhanced Emotional Memory: Cognitive and Neural Mechanisms. Curr Dir Psychol Sci 22: 430–436.

[49] Talmi D, McGarry LM (2012) Accounting for immediate emotional memory enhancement. J Mem Lang 66: 93–108.

[50] Schupp HT, Junghofer M, Weike AI, Hamm AO (2003) Emotional facilitation of sensory processing in the visual cortex. Psychol Sci 14: 7–13. 12564747 10.1111/1467-9280.01411

[51] Todd RM, Talmi D, Schmitz TW, Susskind J, Anderson AK (2012) Psychophysical and neural evidence for emotion-enhanced perceptual vividness. J Neurosci 32: 11201–11212. 22895705 10.1523/JNEUROSCI.0155-12.2012 PMC3449277

[52] Schaefer A, Philippot P (2005) Selective effects of emotion on the phenomenal characteristics of autobiographical memories. Memory 13: 148–160. 15847227 10.1080/09658210344000648

[53] Sharot T, Delgado MR, Phelps EA (2004) How emotion enhances the feeling of remembering. Nat Neurosci 7: 1376–1380. 15558065 10.1038/nn1353

[54] Andreano JM, Cahill L (2006) Glucocorticoid release and memory consolidation in men and women. Psychol Sci 17: 466–470. 16771794 10.1111/j.1467-9280.2006.01729.x

[55] Lupien SJ, McEwen BS (1997) The acute effects of corticosteroids on cognition: integration of animal and human model studies. Brain Res Brain Res Rev 24: 1–27. 9233540 10.1016/s0165-0173(97)00004-0

[56] Gasbarri A, Arnone B, Pompili A, Marchetti A, Pacitti F, et al (2006) Sex-related lateralized effect of emotional content on declarative memory: an event related potential study. Behav Brain Res 168: 177–184. 16443292 10.1016/j.bbr.2005.07.034

[57] Cahill L, McGaugh JL (1998) Mechanisms of emotional arousal and lasting declarative memory. Trends Neurosci 21: 294–299. 9683321 10.1016/s0166-2236(97)01214-9

[58] Lupien SJ, Wilkinson CW, Briere S, Menard C, Ng Ying Kin NM, et al (2002) The modulatory effects of corticosteroids on cognition: studies in young human populations. Psychoneuroendocrino 27: 401–416. 10.1016/s0306-4530(01)00061-0 11818174

[59] Sonnemans J, Frijda NH (1995) The determinants of subjective emotional intensity. Cognition Emotion 9: 483–506.

[60] Bradley MM, Lang PJ (1994) Measuring emotion: The self-assessment manikin and the semantic differential. J Behav Ther Exp Psy 25: 49–59. 10.1016/0005-7916(94)90063-9 7962581

[61] Schaefer A, Nils X, Sanchez X, Philippot P (2010) Assessing the effectiveness of a large database of emotion-eliciting films: A new tool for emotion researchers. Cognition Emotion 24: 1153–1172.

[62] Kensinger EA (2009) Remembering the details: Effects of emotion. Emot Rev 1: 99–113. 19421427 10.1177/1754073908100432 PMC2676782

[63] Mickley Steinmetz KR, Kensinger EA (2009) The effects of valence and arousal on the neural activity leading to subsequent memory. Psychophysiology 46: 1190–1199. 19674398 10.1111/j.1469-8986.2009.00868.x PMC2875869

[64] Mather M, Sutherland MR (2011) Arousal-biased competition in perception and memory. Perspect Psychol Sci 6: 114–133. 21660127 10.1177/1745691611400234 PMC3110019

[65] Mather M, Nesmith K (2008) Arousal-enhanced location memory for pictures. J Mem Lang 58: 449–464. 19190722 10.1016/j.jml.2007.01.004 PMC2390873

[66] Mather M, Knight M (2005) Goal-directed memory: the role of cognitive control in older adults' emotional memory. Psychol Aging 20: 554–570. 16420131 10.1037/0882-7974.20.4.554

[67] Rugg MD, Yonelinas AP (2003) Human recognition memory: A cognitive neuroscience perspective. Trends Cogn Sci 7: 313–319. 12860190 10.1016/s1364-6613(03)00131-1

[68] Park H, Uncapher MR, Rugg MD (2008) Effects of study task on the neural correlates of source encoding. Learn Memory 15: 417–425. 10.1101/lm.878908 PMC2414252 18511693

[69] Nyhus E, Curran T (2009) Semantic and perceptual effects on recognition memory: Evidence from ERP. Brain Res 1283: 102–114. 19505439 10.1016/j.brainres.2009.05.091 PMC2748123

[70] Kensinger EA, Corkin S (2003) Effect of negative emotional content on working memory and long-term memory. Emotion 3: 378–393. 14674830 10.1037/1528-3542.3.4.378

[71] Awh E, Vogel EK, Oh SH (2006) Interactions between attention and working memory. Neuroscience 139: 201–208. 16324792 10.1016/j.neuroscience.2005.08.023

[72] De Fokert Jw, Rees G, Frith CD, Lavie N (2001) The role of working memory in visual selective attention. Science 291: 1803–1806. 11230699 10.1126/science.1056496

