# Supplementary material for: The Effects of a Distracting N-Back Task on Recognition Memory Are Reduced by Negative Emotional Intensity
Source: PLoS One. 2014 Oct 16;9(10):e110211. doi: 10.1371/journal.pone.0110211 (PMC4199670; doi:10.1371/journal.pone.0110211)
Supplement: File S1 — Supplementary analyses. Further analyses were carried out to explore the data on confidence ratings and to assess the separate effects of valence, arousal, and study-test interval on memory performance. (DOCX) [file pone.0110211.s001.docx]

## *Supplementary analyses for Buratto et al.* “The effects of a distracting *N*-back task on recognition memory are reduced by negative emotional intensity”.

These analyses explore a number of questions:

1) Does our manipulation have an effect on confidence ratings?

2) Can the effects of arousal and valence be disentangled?

3) Does a cross-experiment ANOVA reveal any effects of study-test interval?

Question (1) is tackled with participant-level analyses, and question (2) with stimulus-level, regression-based moderation analyses. These analyses are presented below separately for Experiments 1 and 2. Question (3) is addressed at the end with a series of cross-experiment analyses.

## Experiment 1

#### Data analyses

***Participant-level analyses.*** Memory data was analysed using mean confidence ratings and standard recognition measures (hit rates, false-alarm rates, and *Pr*). Mean confidence ratings ranged from 1 (all pictures judged *absolutely sure new*) to 6 (all pictures judged *absolutely sure old*). Hit rate (*HR*) is the proportion of “Old” responses (options “4”, “5”, or “6” in the 6-point confidence scale) given to studied (*Old*) pictures. False-alarm rate (*FAR*) is the proportion of “Old” responses to unstudied (*New*) pictures. *Pr* is the difference between hit rates and false-alarm rates (*Pr* = *HR* – *FAR*).

***Stimulus-level analyses.*** Memory data was also analysed with regression-based tests [[1](#_ENREF_1)]. In this type of analysis, data associated with each picture is the main unit of analysis. This approach has been previously applied to memory data [[2](#_ENREF_2), [3](#_ENREF_3)]. Here the effect of cognitive depletion was estimated for each picture with two separate difference scores: (*i*) the difference between hit rates for each picture in the 0-Back and the 3-Back conditions, which provides a measure of the effect of the 3-Back condition on correct recognition (hits); (*ii*) the difference in *Pr* for each picture between conditions, which provides a measure of the effect of the 3-Back condition on overall picture discriminability (*HR – FAR*). Following Judd et al. [[1](#_ENREF_1)], these difference scores were regressed on mean-centered valence and arousal ratings for each picture, along with measures of picture brightness, contrast and spatial frequency.

#### Results

#### Confidence ratings

A mixed-design ANOVA (Analysis of Variance) on confidence ratings to old pictures was conducted with Picture Type (High, Medium, Low emotional intensity) as a within-subject independent variable and Secondary Task (0-Back, 3-Back) as a between-subject independent variable. The analysis revealed main effects of Picture Type [*F*(2,72) = 108.03, *P <* .001 , $\eta_{p}^{2}$ = .75] and Secondary Task [*F*(1,36) = 7.98, *P <* .01 , $\eta_{p}^{2}$ = .21]. Pairwise contrasts yielded significant differences between High (*M_Conf_* = 5.21, *SE* = 0.07), MED (*M_Conf_* = 4.45, *SE* = 0.11) and Low intensity pictures (*M_Conf_* = 4.08, *SE* = 0.10; *Ps <* .001). Confidence ratings were higher in the 0-Back condition (*M_Conf_* = 4.85, *SE* = 0.12) than in the 3-Back condition (*M_Conf_* = 4.32, *SE* = 0.12, *P* < .01). More importantly, the interaction was significant [*F*(2,72) = 3.47, *P =* .04 , $\eta_{p}^{2}$ = .09]. Planned contrasts showed that the difference in confidence ratings between the N-Back tasks was greater for Medium and Low intensity pictures (*t*s > 2.63, *P*s ≤ .01, Cohen’s *d*s *>* 0.84, large effect) than for High intensity pictures (*t* = 2.12, *P =* .04, *d* = 0.68, medium effect). These results indicate that the 3-Back task reduced more confidence ratings to old Medium and Low intensity pictures than to High intensity pictures.

A similar ANOVA was carried out on confidence ratings to new pictures (higher ratings to new pictures is a measure of false recognition). Here, only main effects of Picture Type [*F*(2,72) = 23.71, *P <* .001, $\eta_{p}^{2}$ = .40] and Secondary Task [*F*(1,36) = 7.07, *P =* .01, $\eta_{p}^{2}$ = .16] were found. The effect of Picture Type reflected lower mean confidence ratings to High intensity pictures (*M_Conf_* = 1.73, *SE* = 0.08) that to Medium (*M_Conf_* = 2.01, *SE* = 0.08) and Low intensity pictures (*M_Conf_* = 2.10, *SE* = 0.09, *P*s < .001), which did not differ from each other (*P* = .10). The effect of Secondary Task reflected higher ratings (false recognition) in the 3-Back task (*M_Conf_* = 1.75, *SE* = 0.11) than in the 0-Back task (*M_Conf_* = 2.15, *SE* = 0.11, *P* = .01).

To assess the extent to which old pictures were more confidently recognized than new pictures, a difference score was calculated. Mean confidence ratings to new pictures (a measure of incorrect recognition) were subtracted from the mean ratings to old pictures (a measure of correct recognition). The resulting score was then used as the dependent variable in a 3 (Picture Type: High, Medium, Low) × 2 (Secondary Task: 0-Back, 3-Back) mixed-design ANOVA. The analysis revealed main effects of Picture Type [*F*(2,72) = 126.71, *P <* .001 , $\eta_{p}^{2}$ = .78] and Secondary Task [*F*(1,36) = 13.78, *P =* .001 , $\eta_{p}^{2}$ = .28], showing that the difference between confidence ratings was higher for High (*M_ΔConf_* = 3.48, *SE* = 0.13) than for Medium (*M_ΔConf_* = 2.44, *SE* = 0.15) and Low intensity pictures (*M_ΔConf_* = 1.98, *SE* = 0.14, *P*s < .001) and that the rating difference was also higher in the 0-Back (*M_ΔConf_* = 3.10, *SE* = 0.18) than in the 3-Back condition (*M_ΔConf_* = 2.17, *SE* = 0.18, *P* = .001). More interestingly, the interaction was marginally significant [*F*(2,72) = 2.83, *P =* .07 , $\eta_{p}^{2}$ = .07], suggesting that the difference between the N-Back tasks in the confidence ratings’ difference score was higher for Medium and Low intensity pictures (*t*s > 3.69, *Ps ≤* .001, *d*s *>* 1.19) than for High intensity pictures (*t* = 2.60, *P =* .11, *d* = 0.84). The results indicate that cognitive depletion, operationalized as a 3-Back task, affected memory confidence ratings to a greater extent when the task was followed by Medium or Low intensity pictures.

#### Stimulus-level moderation analyses

***Hit-rate difference score****.* Cognitive depletion was estimated for each picture by a difference score (*HR_0-Back_* – *HR_3-Back_*). The score for each picture was obtained by calculating the proportion of “Old” responses to old pictures across participants. The larger the difference score, the stronger the impact of cognitive depletion on memory. Following Judd et al. (2001), this difference score was regressed on the mean-centered arousal ratings available for each picture, with picture properties (brightness, contrast and spatial frequency) added as covariates. Arousal scores significantly predicted an attenuation of the decrease in memory performance from the 0-Back to the 3-Back condition (*β*= –.25, *t* = –3.93, *P <* .001). The same regression model was calculated with mean-centered valence scores instead of arousal. Similar results were obtained, although with a smaller effect size (*β*= –.14, *t* = –2.13, *P =* .03). Finally, we computed a multiple regression model in which mean-centered valence and arousal scores were entered as predictors at the same time, with picture properties as covariates. The regression model was significant [*F*(5, 234) = 4.59, *P =* .001] and both arousal and valence had significant unique effects on cognitive depletion, although arousal yielded a more robust effect size (arousal: *β* = –.49, *t* = –4.03, *P <* .001; valence: *β =* .29, *t =* 2.32, *p* = .02).

These results indicate that arousal moderates the decrease in recognition between the 0-Back and the 3-Back conditions. Highly arousing stimuli were more resilient than less arousing stimuli against the memory decrement generated by the concurrent task.

***Pr difference score.*** Another stimulus-level regression was conducted with a difference score based on *Pr*, providing an estimate of the effect of cognitive depletion on memory that is corrected for false-alarm rates. To obtain this difference score, *Pr* values were first calculated for each picture both in the 0-Back condition (*Pr_0-Back_* = *HR_0-Back_ – FAR_0-Back_*) and in the 3-Back condition (*Pr_3-Back_ = HR_3-Back_ – FAR_3-Back_*). These *Pr* values were then subtracted from one another (*Pr_0-Back_* *– Pr_3-Back_*), and this value was used as the dependent variable. The larger the *Pr* difference score, the stronger the impact of cognitive depletion on memory. This score was then regressed on the mean-centered arousal ratings available for each picture, with picture properties added as covariates. Arousal scores significantly predicted a reduction of the impact of the N-Back task on memory (*β*= –.27, *t* = –4.23, *P <* .001). Similar results were found with valence scores (*β*= –.14, *t* = –2.19, *P =* .03). When entered together in the model, both arousal and valence had unique, significant effects on cognitive depletion, with arousal producing a stronger size [*F*(5, 234) = 5.40, *P <* .001; arousal: *β* = –.55, *t* = –4.52, *P <* .001; valence: *β =* .33, *t =* 2.70, *p* = .01].

These results indicate that arousal moderates the decrease in recognition between the 0-Back and the 3-Back conditions. Highly arousing stimuli were more resilient than less arousing stimuli against the memory decrement generated by the concurrent task.

## Experiment 2

#### Data analyses

Data analyses was the same as in Experiment 1.

#### Results

#### Confidence ratings

As in Experiment 1, a 3 (Picture Type: High, Medium, Low) × 2 (Secondary Task: 0-Back, 3-Back) mixed-design ANOVA was conducted on confidence ratings to old pictures. The analysis revealed main effects of Picture Type [*F*(2,74) = 188.65, *P <* .001 , $\eta_{p}^{2}$ = .84] and Secondary Task [*F*(1,37) = 23.30, *P <* .001 , $\eta_{p}^{2}$ = .39], showing that ratings were higher for High (*M_Conf_* = 4.85, *SE* = 0.08) than for MED (*M_Conf_* = 4.01, *SE* = 0.07) and Low intensity pictures (*M_Conf_* = 3.48, *SE* = 0.07, *P*s < .001) and that ratings were also higher in the 0-Back (*M_Conf_* = 4.42, *SE* = 0.09) than in the 3-bBck condition (*M_Conf_* = 3.81, *SE* = 0.09, *P* < .001). In addition, the ANOVA revealed a marginal interaction between Type and Task [*F*(2,74) = 3.04, *P =* .07 , $\eta_{p}^{2}$ = .08], suggesting that the difference in confidence ratings between the N-Back tasks was greater for Medium and Low intensity pictures (*t*s > 4.75, *P*s < .001, *d*s *>* 1.53) than for High intensity pictures (*t* = 2.64, *P =* .01, *d* = 0.84). Consistent with the results of Experiment 1, these results indicate that the 3-Back task reduced confidence ratings to a greater degree when the old pictures were low in arousal than when they were high in arousal.

A 3 × 2 mixed-design ANOVA was also carried out on confidence ratings to new pictures. No main effects or interactions were found (*F*s < 2.46, *P*s > .11).

Finally, a 3 × 2 mixed-design ANOVA was carried out on the difference between confidence ratings. The analysis revealed main effects of Picture Type [*F*(2,74) = 155.91, *P <* .001 , $\eta_{p}^{2}$ = .81] and Secondary Task [*F*(1,37) = 8.48, *P ,* .01 , $\eta_{p}^{2}$ = .19]. The results showed that the difference between confidence ratings was higher for High (*M_ΔConf_* = 2.55, *SE* = 0.14) than for Medium (*M_ΔConf_* = 1.70, *SE* = 0.12) and Low intensity pictures (*M_ΔConf_* = 1.06, *SE* = 0.08, *P*s < .001) and that the rating difference was also higher in the 0-Back (*M_ΔConf_* = 2.08, *SE* = 0.15) than in the 3-back condition (*M_CΔConf_* = 1.47, *SE* = 0.15, *P* < .01). The interaction term was not significant (*F* = 0.60, *P* = .55).

#### Stimulus-level moderation analyses

***Hit-rate difference score.*** As in Experiment 1, a difference score was created for each picture’s hit rates and this score was regressed on each picture’s mean-centered arousal ratings, with brightness, contrast and spatial frequency as covariates. As expected, arousal scores significantly predicted a reduction of the concurrent task’s detrimental effect on memory (*β =* –.25, *t =* –4.61, *P <* .001). The same regression model was calculated replacing mean-centered arousal scores with mean-centered valence scores and similar results were obtained (*β =* –.24, *t =* –4.42, *P <* .001). The regression model including both arousal and valence as regressors was also significant [*F*(5, 314) = 5.16, *P <* .001]. The individual contributions of arousal and valence, however, were not significant (arousal: *β* = –.17, *t* = –1.48, *P* = .14; valence: *β* = –.09, *t* = –0.76, *P* = .45).

***Pr difference score.*** As in Experiment 1, additional stimulus-level regressions were conducted on a difference score based on *Pr*. As expected, arousal scores significantly predicted a reduction of the concurrent task’s detrimental effect on memory (*β =* –.15, *t =* –2.63, *P =* .01). A similar effect was found with valence scores (*β =* –.15, *t =* –2.60, *P =* .01). When entered together in the regression, however, arousal and valence scores were not significant (|*β|*s < .08, |*t*|s < 1, *P*s > .49). The model as a whole was only marginally significant, *F*(5, 314) = 2.09, *P =* .07.

Experiment 2 replicated the results of Experiment 1. Negative stimuli with high levels of emotional intensity were relatively more protected against cognitive depletion than neutral or negative pictures with moderate levels of intensity. This pattern of results was found when using both standard analyses (ANOVAs on *Pr*) and stimulus-level moderation analyses. However, the unique effect of arousal (i.e., independent of valence) as a moderator of cognitive depletion found in experiment 1 was not replicated in experiment 2.

The main difference between Experiments 1 and 2 was the study-test lag, which was increased from 4 hours in the former to 2 days in the latter. Previous research has shown that arousal effects on recognition memory tend to increase with longer retention intervals involving periods of sleep [[4](#_ENREF_4), [5](#_ENREF_5)]. It was thus expected that, if anything, the effects found in Experiment 1 would be amplified in Experiment 2. That was not the case.

#### Cross-experimental analyses

To assess more directly the role of retention interval on the differential effect of cognitive depletion on memory for emotional stimuli, we analysed together the data from Experiments 1 and 2, with *experiment* entered as a between-subject, independent variable. One caveat, however, is that the differences between Experiments 1 and 2 are not restricted to retention interval (for instance, the number of stimuli was larger in Experiment 2). Two sets of analyses were conducted, one with confidence ratings and the other with proportion of “Old” responses.

***Confidence ratings.*** A 3 (Picture Type: High, Medium, Low) × 2 (Secondary Task: 0-Back, 3-Back) × 2 (Experiment: 1, 2) mixed-design ANOVA conducted on confidence ratings to old pictures revealed main effects of Picture Type [*F*(2,146) = 286.45, *P <* .001 , $\eta_{p}^{2}$ = .80; High: *M_Conf_* = 5.03 , *SE* = 0.05; Medium: *M_Conf_* = 4.23 , *SE* = 0.07; Low: *M_Conf_* = 3.78 , *SE* = 0.06, *P*s < .001], Secondary Task [*F*(1,73) = 28.90, *P <* .001 , $\eta_{p}^{2}$ = .28; 0-Back: *M_Conf_* = 4.63 , *SE* = 0.08; 3-Back: *M_Conf_* = 4.06 , *SE* = 0.08], and Experiment [*F*(1,73) = 19.23, *P <* .001 , $\eta_{p}^{2}$ = .21; Experiment 1: *M_Conf_* = 4.58 , *SE* = 0.08; Experiment 2: *M_Conf_* = 4.11 , *SE* = 0.08]. The cross-experimental ANOVA also revealed a significant interaction between Type and Task [*F*(2,146) = 6.35, *P <* .01 , $\eta_{p}^{2}$ = .08], showing that the difference in confidence ratings between the 0-Back and the 3-Back tasks was higher for Medium and Low intensity pictures (*t*s > 4.68, *P*s < .001, *d*s *>* 1.08) than for High intensity pictures (*t* = 3.22, *P =* .002, *d* = 0.73).

A similar ANOVA was conducted on confidence ratings to new pictures. The results revealed a main effect of Picture Type [*F*(2,146) = 16.06, *P <* .001 , $\eta_{p}^{2}$ = .18; High: *M_Conf_* = 2.0103 , *SE* = 0.06; Medium: *M_Conf_* = 2.16 , *SE* = 0.06; Low: *M_Conf_* = 2.26 , *SE* = 0.06, *P*s < .01], a marginal main effect of Secondary Task [*F*(1,73) = 3.17, *P =* .08 , $\eta_{p}^{2}$ = .04; 0-Back: *M_Conf_* = 2.05 , *SE* = 0.08; 3-Back: *M_Conf_* = 2.24 , *SE* = 0.08], and a main effect of Experiment [*F*(1,73) = 13.0, *P =* .001 , $\eta_{p}^{2}$ = .15; Experiment 1: *M_Conf_* = 1.95 , *SE* = 0.08; Experiment 2: *M_Conf_* = 2.34 , *SE* = 0.08]. The ANOVA also revealed a significant interaction between Type and Experiment [*F*(2,146) = 5.67, *P <* .01 , $\eta_{p}^{2}$ = .07], indicating that the rise in (incorrect) confidence ratings between Experiments 1 and 2 was larger for High intensity pictures (*t* = –4.41, *P* < .001, *d =* 1.00) than for Medium and Low intensity pictures (|*t|*s < 2.62, *P*s > .01, *d*s *<* 0.60). A marginal interaction between Task and Experiment was also found [*F*(1,73) = 3.40, *P =* .07 , $\eta_{p}^{2}$ = .04], indicating that there was a slightly larger increase in ratings between 0-Back and 3-Back tasks in Experiment 1 than in Experiment 2.

A third ANOVA was carried out across experiments, this time using a difference score (mean ratings to old pictures minus mean ratings to new pictures) as the dependent variable. The analysis revealed main effects of Picture Type [*F*(2,146) = 278.08, *P <* .001 , $\eta_{p}^{2}$ = .79; High: *M_ΔConf_* = 3.02 , *SE* = 0.09; Medium: *M_ΔConf_* = 2.07 , *SE* = 0.10; Low: *M_ΔConf_* = 1.52 , *SE* = 0.08, *P*s < .001], Secondary Task [*F*(1,73) = 22.34, *P <* .001 , $\eta_{p}^{2}$ = .23; 0-Back: *M_ΔConf_* = 2.59 , *SE* = 0.12; 3-Back: *M_ΔConf_* = 1.82 , *SE* = 0.11] and Experiment [*F*(1,73) = 28.18, *P <* .001 , $\eta_{p}^{2}$ = .28; Experiment 1: *M_ΔConf_* = 2.64 , *SE* = 0.12; Experiment 2: *M_ΔConf_* = 1.77 , *SE* = 0.11]. The ANOVA also yielded a marginal interaction between Type and Task [*F*(2,146) = 2.50, *P =* .08 , $\eta_{p}^{2}$ = .03], suggesting that the difference between the N-Back tasks in the confidence ratings’ difference score was slightly higher for Medium and Low intensity pictures (*t*s > 4.28, *Ps <* .001, *d*s *>* 0.97) than for High intensity pictures (*t* = 2.90, *P =* .005, *d* = 0.67). The results indicate that the 3-Back task affected memory confidence ratings to a greater extent when the task was followed by low-arousal than by high-arousal pictures.

Overall, the cross-experimental analyses of confidence ratings showed that ratings to old pictures decreased and those to new pictures increased between Experiments 1 and 2. This is not surprising, as the retention interval and the number of stimuli were larger in Experiment 2 than in Experiment 1. More importantly for the conclusions of this study, there were no significant three-way interactions between Experiment (taken as a measure of retention interval), Picture Type (a measure of stimulus emotionality) and Secondary Task (a measure of cognitive depletion). In other words, increasing the retention interval from 4 hours to 2 days did not significantly affect the relative memory protection provided by high-arousal pictures under conditions of high cognitive load.

***Proportion of “old” responses****.* A similar set of ANOVAs was conducted using the proportion of “Old” responses as the dependent variable. When the analysis was restricted to old pictures, the dependent variable was a hit rate. When the analysis was restricted to “Old” responses to new pictures, the dependent variable was a false-alarm rate.

*Hits*: A 3 (Picture Type: High, Medium, Low) × 2 (Secondary Task: 0-Back, 3-Back) × 2 (Experiment: 1, 2) mixed-design ANOVA conducted on hit rates revealed main effects of Picture Type [*F*(2,144) = 281.35, *P <* .001 , $\eta_{p}^{2}$ = .80; HighHIGH: *M_HR_* = .84 , *SE* = 0.01; Medium: *M_HR_* = .65 , *SE* = 0.01; Low: *M_HR_* = .54 , *SE* = 0.02, *P*s < .001], Secondary Task [*F*(1,72) = 44.43, *P <* .001 , $\eta_{p}^{2}$ = .38; 0-Back: *M_HR_* = .75 , *SE* = 0.02; 3-Back: *M_HR_* = .60, *SE* = 0.02], and Experiment [*F*(1,72) = 27.94, *P <* .001 , $\eta_{p}^{2}$ = .28; Experiment 1: *M_HR_* = .74 , *SE* = 0.02; Experiment 2: *M_HR_* = .62 , *SE* = 0.02]. The cross-experimental ANOVA also revealed significant interactions between Type and Task [*F*(2,144) = 16.02, *P <* .001 , $\eta_{p}^{2}$ = .18] and between Type and Experiment [*F*(2,146) = 13.48, *P <* .001 , $\eta_{p}^{2}$ = .16]. The first interaction shows that the hit-rate decrease between 0-Back and 3-Back task was larger for Medium and Low intensity pictures (*t*s > 5.58, *P*s < .001, *d*s *>* 1.25) than for High intensity pictures (*t* = 3.11, *P =* .003, *d* = 0.62).

Another 3 × 2 × 2 ANOVA was conducted, this time using false-alarm rate as the dependent variable. The only significant result was an interaction between Picture Type and Experiment [*F*(2,144) = 8.17, *P <* .001 , $\eta_{p}^{2}$ = .10], suggesting that the rise in false-alarm rate between Experiments 1 and 2 was larger for High intensity pictures (*t* = –3.05, *P* = .003, *d =* 0.70) than for Medium and Low intensity pictures (|*t|*s < 0.35, *P*s > .73, *d*s *<* 0.001).

Finally, a three-way ANOVA (3 × 2 × 2) was carried out on *Pr* (the difference between hit rates and false-alarm rates, which provides a measure of memory discriminability). The analysis yielded main effects of Picture Type [*F*(2,144) = 224.22, *P <* .001 , $\eta_{p}^{2}$ = .76; High: *M_Pr_* = .70 , *SE* = 0.02; Medium: *M_Pr_* = .51 , *SE* = 0.02; Low: *M_Pr_* = .40 , *SE* = 0.02, *P*s < .001], Secondary Task [*F*(1,72) = 25.14, *P <* .001 , $\eta_{p}^{2}$ = .26; 0-Back: *M_Pr_* = .62 , *SE* = 0.02; 3-Back: *M_Pr_* = .46 , *SE* = 0.02] and Experiment [*F*(1,72) = 22.71, *P <* .001 , $\eta_{p}^{2}$ = .24; Experiment 1: *M_Pr_* = .61 , *SE* = 0.02; Experiment 2: *M_Pr_* = .46 , *SE* = 0.02]. The ANOVA also produced a highly significant interaction between Type and Task [*F*(2,144) = 12.65, *P <* .001 , $\eta_{p}^{2}$ = .15], showing that the drop in memory discriminability between 0-Back and 3-Bak tasks was larger for Medium and Low intensity pictures (*t*s > 4.89, *Ps <* .001, *d*s *>* 1.12, large effect) than for High intensity pictures (*t* = 1.97, *P =* .05, *d* = 0.41, small effect). The result shows that the 3-Back task affected recognition memory to a greater extent when the task was followed by low-arousal than by high-arousal pictures. Aside from its main effect on discriminability, *Experiment* (retention interval) did not interact with the other factors (*F*s < 1.9, *P*s > .15)

In sum, the cross-experimental analyses on the proportion of “Old” responses showed a reduction in overall discriminability between Experiments 1 and 2 (hit rates decreased and false-alarms increased). More importantly, there were no significant three-way interactions between Picture Type, Secondary Task, and Experiment, suggesting that the longer retention interval in Experiment 2 (2 days) relative to Experiment 1 (4 hours) did not affect the relative memory protection provided by high-arousal pictures under conditions of high cognitive load.

It is however important to note that these cross-experiment analyses are limited by the fact that (1) the experiments are not identical in the number of stimuli and are thus not fully comparable; (2) although this was not the goal of our experiments, a full test of the effects of study-test interval on recognition performance should ideally include an immediate test condition (no delay between the end of the study phase and the beginning of the test phase).

## References

1. Judd CM, Kenny DA , McClelland GH (2001) Estimating and testing mediation and moderation in within-subject designs. Psychol Methods 6: 115-134.

2. Pottage CL, Schaefer A (2012) Visual attention and emotional memory: Recall of aversive pictures is partially mediated by concurrent task performance. Emotion 12: 33-38.

3. Talmi D, Schimmack U, Paterson T, Moscovitch M (2007) The role of attention and relatedness in emotionally enhanced memory. Emotion 7: 89-102.

4. Sharot T, Phelps EA (2004) How arousal modulates memory: Disentangling the effects of attention and retention. Cogn Affect Behav Neurosci 4: 294-306.

5. Payne JD, Stickgold R, Swanberg K, Kensinger EA (2008) Sleep preferentially enhances memory for emotional components of scenes. Psychol Sci 19: 781-788.
